# Human Metapneumovirus Antagonism of Innate Immune Responses

**DOI:** 10.3390/v4123551

**Published:** 2012-12-07

**Authors:** Deepthi Kolli, Xiaoyong Bao, Antonella Casola

**Affiliations:** 1 Departments of Pediatrics, University of Texas Medical Branch at Galveston, Texas, USA; E-Mail: dekolli@utmb.edu (D.K.); xibao@utmb.edu (X.B.); 2 Microbiology and Immunology, University of Texas Medical Branch at Galveston, Texas, USA; 3 Sealy Center for Molecular Medicine, University of Texas Medical Branch at Galveston, Texas, USA

**Keywords:** metapneumovirus, viral proteins, innate immune system, interferon antagonism

## Abstract

Human metapneumovirus (hMPV) is a recently identified RNA virus belonging to the *Paramyxoviridae* family, which includes several major human and animal pathogens. Epidemiological studies indicate that hMPV is a significant human respiratory pathogen with worldwide distribution. It is associated with respiratory illnesses in children, adults, and immunocompromised patients, ranging from upper respiratory tract infections to severe bronchiolitis and pneumonia. Interferon (IFN) represents a major line of defense against virus infection, and in response, viruses have evolved countermeasures to inhibit IFN production as well as IFN signaling. Although the strategies of IFN evasion are similar, the specific mechanisms by which paramyxoviruses inhibit IFN responses are quite diverse. In this review, we will present an overview of the strategies that hMPV uses to subvert cellular signaling in airway epithelial cells, the major target of infection, as well as in primary immune cells.

## 1. Human Metapneumovirus (hMPV): A Recently Discovered Human Viral Pathogen

**Figure 1:**
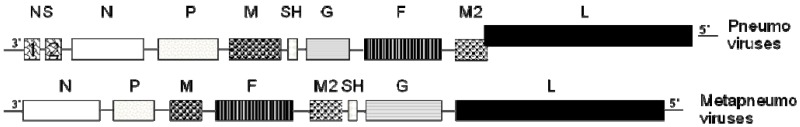
Genomic organization of *Pneumovirinae*.

The *Paramyxoviridae* family includes enveloped, negative-sense, single-stranded RNA viruses, which are major and ubiquitous disease causing pathogens of humans and animals [1]. Among them are important viruses that cause acute respiratory morbidity, particularly in infancy, elderly and in immunocompromised subjects of any age. The family is taxonomically divided into two subfamilies, the *Paramyxovirinae*, with five genera, and the *Pneumovirinae,* which includes two genera (Table 1). The classification of these viruses is based on their genome organization, morphological and biological characteristics, and sequence relationship of the encoded proteins. The pneumoviruses can be distinguished from the *Paramyxovirinae* members morphologically because they contain narrower nucleocapsids [1]. In addition, pneumoviruses have differences in genome organization, the number of encoded proteins and an attachment protein that is different from that of members of the subfamily *Paramyxovirinae*. There are two genera in the *Pneumovirinae *family, the *Pneumovirus *genus that includes human and bovine respiratory syncytial virus (RSV) and the *Metapneumovirus *genus that includes human metapneumovirus (hMPV) and avian metapneumovirus (APV) (Table 1). Human RSV encodes 11 separate proteins, while hMPV encodes nine proteins that generally correspond to those of RSV, except that hMPV lacks the non-structural proteins NS1 and NS2 and the gene order is different from that of pneumoviruses (Figure 1).

**Table 1:** Representative members of the *Paramyxoviridae* family

Subfamily	Genus	Virus
*Paramyxovirinae*		
	Henipavirus	Hendravirus
		Nipah virus
	Morbillivirus	Measles virus (MeV)
	Respirovirus	Sendai virus (SeV)
		Human parainfluenza virus type 1 (hPIV1)
		Human parainfluenza virus type 3 (hPIV3)
		Bovine parainfluenza virus type 3 (BPIV3)
	Rubulavirus	Parainfluenza virus type 5 (PIV5)
		Mumps virus (MuV)
		Human parainfluenza virus type 2 (hPIV2)
*Pneumovirinae*		
	Pneumovirus	Human respiratory syncytial virus (hRSV)
		Bovine respiratory syncytial virus (BRSV)
	Metapneumovirus	Avian pneumovirus (APV)
		Human metapneumovirus (hMPV)

Since its first identification in 2001, hMPV has been isolated from individuals of all ages with acute respiratory tract infection worldwide [2]. Virtually, all children older than five years show 100% serologic evidence of infection [3]. Around 12% of all respiratory tract infections in children are caused by hMPV, second only to RSV [2,4,5,6]. HMPV also accounts for 10% of all hospitalizations of elderly patients with respiratory tract infections and it has been isolated from respiratory samples of a single winter season as often as parainfluenza [7]. Phylogenetic analysis of strains from many countries demonstrates two distinct hMPV genotypes, A and B, which can be divided in two subgroups: A1, A2, B1 and B2 [2,4]. The clinical features associated with hMPV in children are similar to those of RSV. hMPV is associated with both upper and lower respiratory tract infections. Fever, cough, tachypnea, wheezing and hypoxia are frequently observed in infected children. Chest radiographs demonstrate focal infiltrates and peribronchial cuffing. Many children have a clinical syndrome consistent with bronchiolitis. A significant proportion of symptomatic children who tested positive for hMPV had co-morbidities such as a history of prematurity, chronic lung disease or complex congenital heart diseases [8]. These findings suggest that the populations of children prone to severe RSV disease may be also prone to hMPV disease. Although RSV and hMPV share similar clinic features, hMPV induces a different spectrum of immune mediators compared to RSV [9,10,11], suggesting that the host cell responses and likely the pathogenesis of lung disease are viral specific.

## 2. Pattern Recognition Receptors (PRRs) in hMPV-Induced Signaling

The innate immune response represents a critical component of the host defense against viruses and is coordinated at the cellular level by activation of transcription factors that regulate the expression of inducible gene products with antiviral and/or inflammatory activity. Viruses contain conserved structural moieties, known as pathogen associated molecular pattern (PAMPs), that are recognized by several families of PRRs, in particular Toll-Like Receptors (TLR) and RNA helicases. Their relative contribution in virus-triggered cellular signaling is stimulus- and cell-type-dependent (Reviewed in [12]). So far, 10 members of TLRs have been identified in humans, and 13 in mice. Among those, TLR3, 4, 7, 8 and 9 have been shown to be more commonly involved in the innate response to viral infections [13,14]. TLR3 recognizes double-stranded RNA (ds-RNA) that is produced during viral replication [12]. Recently, RSV has been shown to relocate TLR3 from endosomes to cytoplasmic membrane in infected airway epithelial cells, resulting in increased chemokine production after an initial phase that is dependent on RSV replication [15,16]. However, we did not find a similar role of TLR3 in hMPV-induced cellular signaling either in airway epithelial cells [17] or in primary immune cells, such as dendritic cells (DCs) [18].

Several viral envelop proteins, including RSV F protein and proteins of mammary tumor virus, murine leukemia virus, vesicular stomatitis virus [19,20,21], and more recently Ebola virus [22], have been shown to activate TLR4 in primary immune cells. Similar to LPS, the primary ligand of TLR4, RSV F protein requires the presence of CD14 and MD-2 for signaling [23,24]. TLR4 signaling has been shown to play an important role in controlling paramyxovirus infection. TLR4-deficient mice challenged with RSV exhibited impaired natural killer (NK) cell and CD14+ cell pulmonary trafficking, diminished NK cell function, and impaired IL-12 induction, in addition to impaired RSV clearance [20]. In a model of alveolar macrophage depletion of TLR4-defective C3H/HeJ mice, we have shown that the early NF-κB response that occurs in the lung after RSV infection, is dependent upon alveolar macrophages and TLR4 [25]. Furthermore, both TLR4 and the adaptor molecule MyD88 have been shown to be required for optimal protection against viral challenge in a mouse model of RSV infection [26]. Our recent investigations have shown that down regulation of TLR4 expression in human DCs or lack of functional TLR4 in mouse bone marrow-derived DCs result in significantly reduced expression of hMPV-induced cytokine, chemokine, and type I IFN secretion, indicating an important role of this TLR in the activation of cellular signaling following hMPV infection [18]. In addition, mice lacking TLR4 showed less clinical disease, significantly lower levels of cytokines and chemokines, compared to the wild type. Accordingly, inflammatory cell recruitment in the BAL, lungs, as well as in lymph nodes, was also significantly reduced [27]. These results indicate that TLR4 is important for activation of the innate immune response to hMPV infection, however, it also contributes to disease pathogenesis.

TLR7 and 8 share highest homology to each other among the TLR family and both of them recognize single-stranded RNA (ss-RNA) [28,29], while TLR9 recognize viral CpG DNA motif [30,31]. In case of hMPV, it has been shown that induction of cytokines and type I Interferon (IFN) is TLR7-dependent [32], using TLR7-deficient mice and TLR7 specific oligonucleotide-based inhibitor ISS661. While a similar requirement in RSV infection of pDCs has not been demonstrated yet, recently a critical role for TLR7 and MyD88 in the recognition and development of innate inflammatory responses necessary to limit pneumovirus infection has been reported [33]. No direct involvement of TLR9 in innate immune cellular signaling in response to either RSV or hMPV has been reported yet.

**Figure 2:**
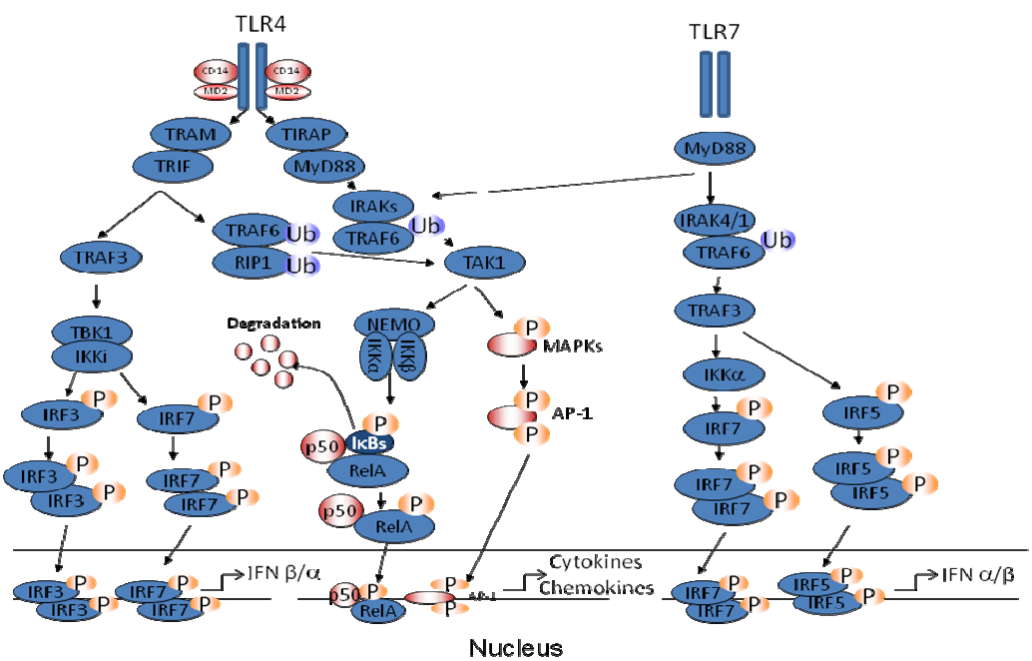
**Toll-Like Receptors (TLR) signaling pathway involved in hMPV-induced gene expression in primary immune cells.** Upon binding of their specific viral PAMP, TLR4 and 7 lead to activation of NF-κB- and IRF-dependent gene expression by engaging the adaptor MyD88 alone (TLR7) or in combination with TRIF (TLR4). Ub indicates ubiquitination; P indicates phosphorylation.

After recognition of their own PAMPs, following viral infection, TLRs trigger intracellular signaling pathways that are necessary to the induction of inflammatory cytokines, chemokines, as well as type I IFN. Three structural domains, *i.e*., a Leucine Rich Region (LRR) in the N-terminal ectodomain, a transmembrane region, and a Toll/IL-1R resistance (TIR) domain in the intracellular region, are structural hallmarks of all known Toll/TLRs. Differential utilization of four TIR-containing adapter molecules (*i.e.*, MyD88, TIRAP, TRIF, and TRAM) by distinct TLRs leads to activation of downstream signaling pathways, findings based largely on studies in adapter knockout mice. Two major TLR signaling pathways have been identified, *i.e*., one that is MyD88-dependent, and gives rise to strong and early activation of the transcription factor NF-κB, and a TRIF-dependent, MyD88-independent pathway that primarily drives strong activation of IRF-3, with later activation of NF-κB. The MyD88-dependent pathway results in induction of highly NF-κB-dependent, proinflammatory genes (TNF-α, IL-1β, IL-6), while the MyD88-independent pathway leads to gene induction that is highly IRF-3-dependent (IFN-β, RANTES). TLR4 activates both pathways for gene expression, as it is the only TLR that uses both adapter proteins (Figure 2) [13,34,35].

**Figure 3:**
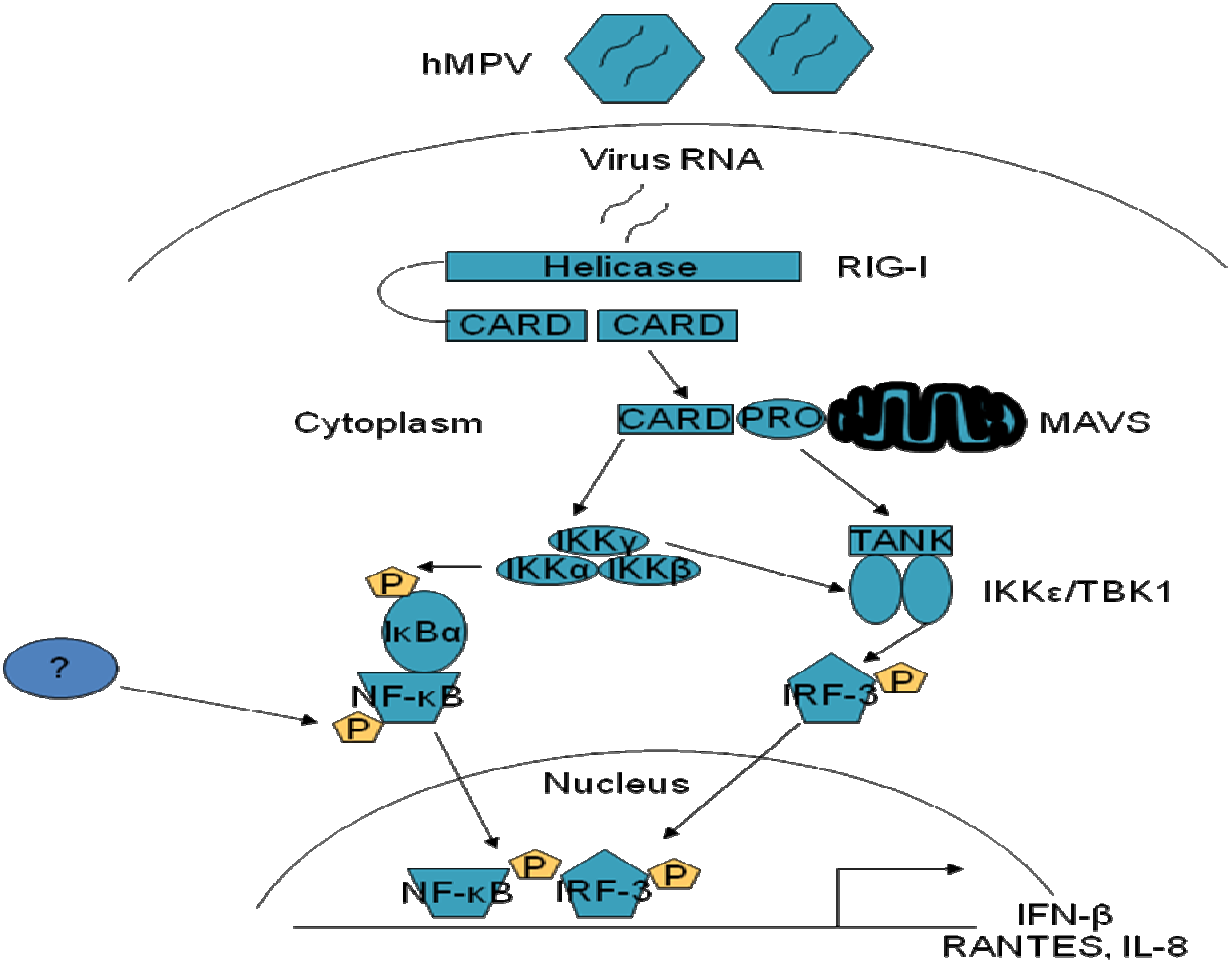
**RIG-I/MAVS signaling pathway regulating hMPV-induced NF-κB and IRF activation in airway epithelial cells.** Production of specific RNA moieties during viral replication leads to activation of the RIG-I-MAVs pathway, which subsequent activation of the IKK complex, which is upstream NF-κB activation, and of the TBK1/IKKβ complex, which regulates IRF-3 activation, leading to proinflammatory/immune gene expression. P indicates phosphorylation; ? indicates the unknown kinase that phosphorylates NF-кB in response to hMPV infection.

Aside from the recognition of viral RNA through the TLRs, two DExD/H box RNA helicases, retinoic acid inducible gene (RIG)-I and melanoma differentiation-associated gene (MDA)-5, have been identified to be essential for IFN induction by several viruses including Newcastle, Sendai, hepatitis C, influenza and RSV [16,36,37,38,39]. Both RIG-I and MDA-5 share two homologous CARD domains and a helicase domain that is required for its interaction with viral RNA [36,40]. The CARD domains of the RNA helicases mediate its interaction with the CARD domain of the mitochondrial protein MAVS (also known as IPS-1/VISA/Cardif), leading to subsequent activation of downstream signaling molecules, such as IRFs, NF-κB and AP-1 [41,42,43,44]. Viral RNA signaling mediated by RIG-I is independent from TLRs, as dominant negative RIG-I does not block TLR-mediated signaling [41,43]. Generation of RIG-I and MDA-5 knock-out mice has identified a major antiviral role for RNA helicases in several cell types, including macrophages and conventional DCs, but not in plasmacytoid DCs (pDCs), which instead require TLR7 and/or TLR9 to mount an effective antiviral response (Reviewed in [44]). We have recently shown that hMPV infection of airway epithelial cells induces the expression RIG-I and MDA-5 and that RIG-I, but not MDA-5, plays a fundamental role in hMPV-induced cellular signaling, as inhibition of RIG-I expression significantly decreases activation of IRF and NF-кB transcription factors and production of type I IFN and proinflammatory cytokines and chemokines [17]. MAVS was also necessary for hMPV-induced cellular signaling, as expression of a dominant negative mutant MAVS significantly reduced IFN-β and chemokine gene transcription, in response to hMPV infection. RIG-I-dependent signaling was necessary to induce a cellular antiviral state, as reduction of RIG-I expression resulted in enhanced hMPV replication (Figure 3) [17].

## 3. Inhibition of TLR Signaling

Dendritic cells are professional antigen-presenting cells that can be generally divided into myeloid CD11c^+^ ‘conventional’ DCs (cDCs) or pDCs [45]. Both subsets specialize in detecting viruses and initiating innate and adaptive immune responses that lead to viral elimination or control [46]. Under baseline conditions, these cells reside in the peripheral tissue in an immature, resting state scattered throughout the respiratory mucosal wall [47] and express several receptors for recognizing viruses [48] such as TLRs. These DCs are often the first immune cells to come in contact with infecting viruses after infection, particularly after mucosal exposure, and can became primary targets of infection. After detection, uptake, and degradation of viruses, DCs initiate immune responses via the secretion of interferon, chemokines, and proinflammatory cytokines, and the upregulation of a variety of costimulatory molecules and receptors, a process globally known as cell maturation. The central role of DC in initiating and shaping the immune response, together with their presence at and recruitment to the site of infection and concomitant exposure to infectious virus, makes them obvious candidates for viral manipulation of the host immune response.

We and others have reported that hMPV can infect monocyte-derived DCs (moDCs), resulting in viral antigen expression [10,49,50] and maturation (increased expression of MHC II, CD86) [10,50]. HMPV can also infect pDCs, although less efficiently than moDCs [10]. In recent investigations we have shown that hMPV infection inhibits TLR-dependent signaling both *in vitro* and *in vivo.* Type I IFN production in isolated moDCs, following stimulation with TLR3 and 4 agonists, and in pDCs, in response to a TLR9 agonist, was significantly reduced by hMPV infection in a replication-dependent manner [10,18]. Furthermore, prior infection of BALB/c mice with hMPV completely suppressed IFN-α production induced by intranasal application of poly-ICLC (TLR3 ligand) or a synthetic CpG-ODN (TLR9 ligand) in mice lung [51], indicating that hMPV interferes with one or multiple signal transduction pathways activated in response to TLR stimulation in a variety of cell types.

## 4. Interferon Signaling Antagonism

IFNs are a group of cytokines that activate an array of cellular genes that are critical in restricting viral replication and modulating adaptive immunity. Production of IFNs is an important feature of the host response to viral infections. Type I IFNs (IFN-α and -β) are the key mediators produced by airway epithelial cells infected with paramyxoviruses [36,52,53] including hMPV [54]. Secreted IFN-α/β bind to IFN-α/β receptors (IFNαR) leading to dimerization of the two subunits, IFNαR1 and IFNαR2. IFNαR1 and IFNαR2 then undergo conformational changes resulting in the activation of the Janus tyrosine kinase (Jak)/signal transducer and activator of transcription protein (STAT) pathway [55]. Tyrosine kinase 2 (Tyk2), a kinase belonging to the Jak family, is constitutively bound to IFNAR1. Tyk2 phosphorylates IFNAR1 at tyrosine residue 466 (Y466) and creates a docking site for STAT2 [56]. Subsequently, Tyk2 phosphorylates STAT2 at tyrosine 690 (Y690). Phosphorylation of STAT2 Y690 creates a new docking site for the SH2 domain of STAT1 [57,58], which is subsequently phosphorylated at Y701 by IFNAR2 bound-Jak1 [59]. Phosphorylated STAT1 and STAT2 then dimerize and bind to IRF-9 [60]. This newly formed heterotrimer, known as IFN-stimulated gene factor 3 (ISGF3), translocates into the nucleus to bind ISG gene promoter and activate transcription. ISGs induced by type I IFN signaling typically contain either interferon stimulated response elements (ISRE) or a gamma activated sequence (GAS) elements within their promoters, although there is a clear preference for genes containing an ISRE. Examples of ISRE-containing ISGs are ISG15, Myxovirus (influenza virus) resistance (Mx)1, 2'-5'-oligoadenylate synthetase (OAS)1, IRF-7 and protein kinase R (PKR) [61], while GAS-containing genes are IRF-1, IRF-2, IRF-8 and IRF-9 [62,63]. In addition to activating this canonical Jak/STAT pathway described above, stimulation of the IFNαR also activates several non canonical signaling events such as recruitment and phosphorylation of other STATs [61,62,63] and tyrosine phosphorylation of and activation of insulin receptor substrates 1 (IRS1) and 2 (IRS2) [63].

**Figure 4:**
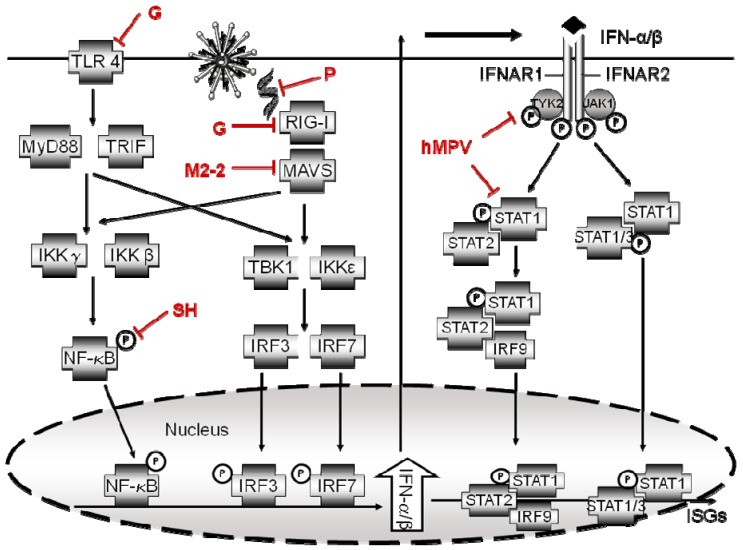
**Schematic diagram of sites of antagonism of cellular signaling and IFN production/responses by hMPV proteins.** Major cellular targets of hMPV proteins in the TLR4, RIG-I/MAVS and type I IFN signaling pathways. P indicates phosphorylation.

As IFN response is critical for a robust innate immune response, almost all mammalian viruses have developed strategies to interfere with IFN production and signaling and to disrupt innate host antiviral factors. These include directly targeting the pathways required for the induction of IFN production, targeting of signaling molecules belonging to the Jak/STAT signaling pathway, and increasing the expression or activity of endogenous cellular key regulators, such as suppressor of cytokine signaling (SOCS) proteins, protein tyrosine phosphatases (PTPs) and protein inhibitor of activated STATs (PIAS) [64,65]. Several members of the *Paramyxovirus* family have been shown to directly target STAT signaling through distinct mechanisms which include proteasomal degradation [66,67,68], sequestration in high-molecular-weight complexes [69,70] and inhibition of nuclear localization of STAT proteins [71]. The first description of hMPV capacity to interfere with IFN signaling came from Harrod *et al. *who tested the ability of hMPV to subvert IFN-α-dependent responses in airway epithelial cells using reporter gene assays. They showed that IFN-α-mediated induction of ISRE-driven luciferase activity was completely abolished in hMPV-infected A549 cells, as well as induction of ISGs, such OAS1, Mx1, RIG-I and MDA-5 [72]. This observation was paralleled by the inhibition of IFN-α-dependent tyrosine phosphorylation and subsequent nuclear translocation of STAT1 in hMPV-infected airway epithelial cells [72]. However, a mechanism responsible for the observed inhibition was not identified. Later, we have shown that hMPV infection affects several steps of the IFN signaling pathway, from inducing degradation of Jak1 and Tyk2 via a ubiquitin-proteasome-dependent pathway, to reducing IFNAR1 surface expression in infected cells, possibly due to increased internalization of the receptor as a result of viral-induced degradation of Tyk2 (Figure 4) [73]. Both phenomena were independent of type I IFN expression, since inhibition was also observed in Vero cells which do not produce type I IFN, and required *de novo* viral gene expression and/or viral RNA replication [73].

## 5. hMPV Proteins Identified as Antagonist of Host Innate Signaling Pathways

Among hMPV encoded proteins, phosphoprotein P, glycoprotein G, small hydrophobic protein (SH) and M2-2 have been shown to modulate hMPV-induced innate immune response, the first line of host defense against invading pathogens [18,32,74,75,76]. A discussion of the inhibitory function for each of these proteins is presented below.

Small hydrophobic (SH) glycoprotein. hMPV SH protein is a type II transmembrane glycoprotein [77]. It is the largest among the pneumoviruses (179 aa for the hMPV isolate CAN97-83 versus 175 aa for APV, 81 aa for BRSV, and 64 aa for HRSV) [77,78]. Even though it is substantially longer than RSV, it has similar characteristics to the one of RSV, including a high percentage of threonine and serine residues and a similar hydrophilicity profile [77,78]. HMPV SH protein is more prone to frequent frameshift and point mutations in culture presumably to give a selective advantage of the clinical isolates in culture [79] and does not appear to be required for virus growth *in vitro*. In fact, a recombinant hMPV virus lacking the SH protein (rhMPV-ΔSH) is viable, grows as well as the wild-type virus in MK2 cells and A549 cells [74,80] and is not significantly attenuated in animal models of infection [74,80,81]. Similar to the SH protein of several members of the *Paramyxoviridae* family such PIV5 and RSV [82,83], and more recently J paramyxovirus (JPV) [84] and mumps virus (MuV) [85], all of which have been shown to inhibit TNF-α-mediated NF-кB signaling, we have also reported an inhibitory role of hMPV SH protein in NF-κB activation [74]. Infection of airway epithelial cells with rhMPV-ΔSH led to increased interleukin 6 (IL-6), IL-8, and MCP-1 secretion, compared to rhMPV-WT [74]. Similarly, BALB/c mice infected with rhMPV-ΔSH showed enhanced production of TNF-α, IL-6, KC and MCP-1, compared to rhMPV-WT [74]. We observed significantly higher induction of IL-8 gene transcription in 293 cells infected with rhMPV-ΔSH compared to rhMPV-WT, and SH protein expression lead to inhibition of TNF-α induced IL-8 promoter activation, confirming a role of SH in inhibition of NF-κB-dependent gene transcription [74]. SH protein of hMPV affected NF-κB-dependent gene transcription by modulating NF-κB transcriptional activity and not by inhibiting nuclear translocation, and therefore the canonical pathway leading to NF-κB activation (Figure 4) [86]. Similar to Rep78 protein of adeno-associated virus type II, which targets PKA activation [87], hMPV SH might inhibit one of the kinases that phosphorylate NF-κB [74]. Our results indicate a possible novel mechanism by which paramyxovirus SH proteins can affect NF-κB activation, in addition to inhibiting TNF-induced NF-κB nuclear translocation, as it has been shown for RSV and PIV5 SH proteins [82,83]. Whether RSV, PIV5 and JPV SH proteins can also affect viral-induced NF-κB post-translational modifications will require further investigation.

Glycoprotein G. hMPV G protein is a type II mucin-like glycosylated protein [77]. The membrane anchor of G protein is proximal to the N terminus and their C terminus is oriented externally. Although the postulated function of G protein is for attachment, it has been shown that hMPV F protein alone is sufficient to mediate attachment and fusion in the absence of other surface proteins including G [80,81,88]. In addition, the interaction of F with cellular integrin receptors is independent of G protein [89], suggesting that G protein plays a minimal role in hMPV attachment. Although lack of G protein does not decrease significantly the ability of hMPV to replicate *in vitro*, a recombinant virus in which the G protein is deleted (rhMPV-ΔG) exhibits reduced replication in the upper and lower respiratory tract of Syrian hamsters and African green monkeys [80,81].

In the past few years, our laboratory has been focusing on the molecular mechanism(s) underlying the attenuation of rhMPV-ΔG. We discovered that hMPV G is an important virulence factor which inhibits cellular signaling both in airway epithelial cells and in primary immune cells [75,90]. The respiratory epithelium represents the principal and primary target site of respiratory viruses, including hMPV. We found that rhMPV-ΔG induces significantly higher amounts of the cytokines, chemokines and type I IFN than hMPV-WT in airway epithelial cells, due to enhanced activation of transcription factors belonging to the NF-κB (e.g. p65 and p50) and IRF families (e.g. IRF-3), demonstrated by increased nuclear translocation and/or phosphorylation [75]. As RIG-I and MAVS are central regulators of hMPV-induced cellular signaling in airway epithelial cells, we investigated whether G targeted the RIG-I/MAVS pathway and, indeed, we found that G protein physically interacts with RIG-I and inhibits RIG-I-, but not MAVS-dependent IFN-β gene transcription (Figure 4) [75].

We have recently investigated the molecular mechanism by which G protein expression inhibits RIG-I activation. We found that the interaction of hMPV G with RIG-I occurs primarily through the CARD domains of its N-terminus, preventing RIG-I association with the adaptor protein MAVS and RIG-I recruitment to mitochondria. HMPV G expression also prevented the interaction between mitochondria and mitochondria-associated membrane (MAM) component of the endoplasmic reticulum (ER), which contains Stimulator of IFN genes (STINGS), an important part of the viral-induced RIG-I/MAVS signaling pathway, leading in the end to the inhibition of cytokine, chemokine and type I interferon (IFN) expression. Mutagenesis analysis showed that hMPV G protein cytoplasmic domain played a major role in the observed inhibitory activity, and recombinant viruses expressing a G protein with amino acid substitution in position 2 and 3 recapitulated most of the phenotype observed with rhMPV-ΔG mutant upon infection of airway epithelial cells [91].

DCs play a pivotal role in shaping antiviral immune responses in the respiratory tract. They can efficiently sense invading pathogens by TLRs and, because of their strategic localization at mucosal sites, are involved in the response to viral infections [92,93]. We have recently demonstrated that moDCs infected with rhMPV-∆G also produce higher levels of type I IFNs, cytokines and chemokines compared with cells infected with rhMPV-WT, suggesting that G protein plays an inhibitory role in viral-induced cellular responses of immune cells as well (Figure 4) [90]. As discussed above, TLR4 plays a major role in hMPV-induced activation of moDCs [90]. In our study, we found that G protein impaired TLR4-dependent signaling, as moDCs infection with rhMPV-∆G inhibited LPS-induced production of cytokine and chemokines significantly less than rhMPV-WT, and treatment of moDCs with purified G protein resulted in significant inhibition of LPS-dependent signaling. Taken together, our results demonstrate that hMPV G protein plays an important role in inhibiting host innate immune responses in dendritic cells, possibly affecting adaptive immune responses as well. This is an important finding, as inadequate TLR stimulation, with subsequent lack of antibody affinity maturation has been recently identified as an important cause of vaccine failure and enhanced disease following administration of the formaline-inactivated RSV vaccine [94].

Inhibition of cellular signaling by surface glycoproteins has been demonstrated for other viruses as well. RSV G protein inhibits cytokine and chemokine secretion, as infection with a recombinant RSV lacking the full-length G protein (rRSV-ΔG) or the soluble part of G protein (rRSV-ΔsG) enhances production of IL-6 and IL-8 in monocytes [95], and IL-8 and RANTES secretion, and ICAM expression in airway epithelial cells [96]. RSV-ΔG also induces more immune mediator production, compared to WT virus, in a mouse model of infection [95]. Similarly, the surface glycoproteins of hantaviruses, in particular, those associated with hemorrhagic pulmonary syndrome (HPS), have been shown to affect IRF-3 activation and IFN production via interaction with RIG-I and TBK-1, a kinase responsible for viral-induced IRF-3 phosphorylation, and to inhibit IFN-mediated cellular responses [97,98]. Taken together, it could be a common feature of surface glycoproteins of enveloped single strand, negative strand RNA viruses to be inhibitory to antiviral signaling, consequently, leading to host immune evasion. 

M2-2 protein. HMPV M2 encodes two overlapping proteins: M2-1 and M2-2. The M2-1 open reading frame (ORF) of strain CAN 97-83 is assumed to start with the first AUG at nucleotide position 14, and encodes a protein of 187 amino acids. The M2-2 ORF possibly initiates with the AUGs at positions 525 and 537, overlapping the M2-1 ORF by 53 or 41 nucleotides, respectively [77,99]. M2-1 protein is not essential for hMPV recovery using the reverse genetic system *in vitro*, in contrast to RSV M2-1 protein, which is essential for full viral replication [99,100,101]. The role of hMPV M2-2 protein in regulating viral RNA synthesis has been confirmed by our group and several others [81,99,102,103].

Similar to rhMPV-∆G, recombinant rhMPV lacking M2-2 (rhMPV-ΔM2-2) is also listed as a live vaccine candidate, as it is attenuated, immunogenic and protective against hMPV challenge in both African green monkeys and hamsters [81,99,102]. Although the attenuation of rhMPV-ΔM2-2 could be explained by decreased viral genome accumulation by M2-2 deletion [99,102,103], we found that other mechanism(s) could be also associated with rhMPV-ΔM2-2 attenuation, as discussed below. We recently discovered that hMPV M2-2 is an important antagonist of host antiviral signaling, therefore, favoring hMPV replication. In fact, ∆M2-2-infected airway epithelial cells produced higher levels of IFN-β and other immune mediators, compared to rhMPV-WT-infected cells [76]. Although the expression of hMPV G protein was impaired due to a reduced ability of rhMPV-ΔM2-2 to replicate, which might contribute indirectly to the enhanced cellular responses observed following infection with the M2-2 deleted mutant, ectopic expression of G protein at levels comparable or higher than the one observed in WT-infected cells only partially reversed the enhancement in cellular responses observed with rhMPV-ΔM2-2, suggesting that M2-2 contributes to hMPV immune evasion as well. Indeed, in reporter gene assays, M2-2 protein, but not other soluble hMPV proteins, inhibited MAVS-induced IFN-β gene transcription, but not the induction mediated by downstream signaling molecules of the RIG-I/MAVS pathway, suggesting that MAVS is the target of M2-2 (Figure 4). Coimmunoprecipitation studies, both in an overexpression system or in the context of viral infection, showed a clear association of M2-2 with MAVS, supporting this concept [76].

We have also identified the domains of M2-2 responsible for the regulation of viral gene transcription, viral replication, and RIG-I-mediated signaling. We found that the first 25 amino acids of M2-2 are critical to promote viral gene transcription, but not involved in the regulation of viral replication and hMPV-induced signaling. In contrast, the domains spanning from amino acid 26 to 69 are dispensable for the regulation of viral gene transcription, but responsible for RIG-I signaling inhibition and viral replication facilitation [76].

In summary, hMPV uses both G and M2-2 protein to target the RIG-I/MAVS pathway. The mechanism by which viruses use two distinct viral proteins to target molecules belonging to the same cellular signaling pathway is becoming recognized as a common strategy to evade host immune defenses. For example, influenza virus uses its NS1 protein to target RIG-I [104,105], and its PB1-F2 and PB2 proteins to inhibit MAVS [106,107]. In case of RSV, NS2 protein of RSV antagonizes the activation of IFN-β transcription by interaction with RIG-I [108], and we recently found that NS1 protein inhibits IFN-β synthesis by associating with RIG-I downstream transcription factor IRF-3 and its transcriptional co-activator CBP [109].

Phosphoprotein (P). The P protein gene of the pneumoviruses, different from the one of paramyxovirinae, is monocistronic, encoding a single polypeptide with a predicted mass of 32.5 kDa [110], although production of multiple forms of phosphoprotein in infected cells has been reported [111]. Similar to other pneumoviruses, hMPV P protein is present within the helical ribonucleoprotein (RNP) complex. It has been shown that hMPV N and P proteins interact together and form cytoplasmic inclusion bodies [112].

Elegant studies conducted by Goutagny *et al* [32], focusing on comparing the innate responses to hMPV A and B strains, showed that hMPV-B failed to elicit a type I IFN response in airway epithelial cells and in human monocytes, despite its ability to infect and replicate as efficiently as hMPV-A. Similar to our reports in epithelial cells [17], Goutagny and coworkers showed that hMPV A1 triggers RIG-I activation to induce type I IFN production while hMPV B1 did not [32]. They showed that in the context of the virus infection, the hMPV B viral RNA is prevented from being sensed by RIG-I and that the P protein is responsible for this inhibition (Figure 4). Since viral RNA is protected from free cellular RNases by RNP complex, proteins within the RNP, especially P protein in hMPV B1 probably could prevent the recognition of the viral RNA by the RIG-I pathway. This strain specific inhibition of RIG-I sensing by P protein has been ascribed to higher levels of expression of the P protein, as well as higher affinity for the RNA or other components of the RNP complex, during hMPV-B infection, compared to hMPV-A [32]. Moreover, the inhibitory effect of the hMPV-B P protein was restricted to the RIG-I pathway, because it did not prevent the induction of IFN-α/β in pDCs, which uses TLR7 pathway. In conclusion, this study suggests that in addition to other structural proteins such as G, SH and M2-2 proteins, RNP complex proteins might also play an important role in inhibition of viral- induced type I IFN expression.

In summary, hMPV antagonizes cellular responses and type I IFN secretion/signaling through a variety of mechanisms which involve the regulation of PRRs (both TLRs and RLR), as well as the key signaling molecules involved in the downstream signaling of IFN pathway such as STAT1, STAT2, Jak1 and Tyk2. The role of this inhibition of type I IFN signaling by hMPV in pathogenesis and severity of infection also needs to be investigated, as inhibition of IFN signaling may affect development of host adaptive immunity, leaving the host susceptible to reinfection. A better understanding of how hMPV inhibits cellular signaling and type I IFN pathway, and its consequences in regard to the innate and adaptive immune responses, is crucial for improving therapeutic approaches and the development of better vaccines against hMPV infection.

## References

[1] Lamb R.A., Kolakofsky D., Knipe D.M., Howley P.M. (2001). Paramyxoviridae: The Viruses and Their Replication. Fundamental Virology.

[2] Principi N., Bosis S., Esposito S. (2006). Human metapneumovirus in paediatric patients. Clin. Microbiol. Infect..

[3] van den Hoogen B.G., de Jong J.C., Groen J., Kuiken T., de Groot R., Fouchier R.A., Osterhaus A.D. (2001). A newly discovered human pneumovirus isolated from young children with respiratory tract disease. Nat. Med..

[4] Kahn J.S. (2006). Epidemiology of human metapneumovirus. Clin. Microbiol. Rev..

[5] Williams J.V., Harris P.A., Tollefson S.J., Halburnt-Rush L.L., Pingsterhaus J.M., Edwards K.M., Wright P.F., Crowe J.E. (2004). Human metapneumovirus and lower respiratory tract disease in otherwise healthy infants and children. N. Engl. J. Med..

[6] Crowe J.E. (2004). Human metapneumovirus as a major cause of human respiratory tract disease. Pediatr. Infect. Dis. J..

[7] Boivin G., Abed L., Pelletier G., Ruel L., Moisan D., Cote' S., Peret T.C., Erdman D.D., Anderson L.J. (2002). Virological features and clinical manifestations associated with the human metapneumovirus, a newly discovered paramyxovirus. J. Infect. Dis..

[8] Esper F., Boucher D., Weibel C., Martinello R.A., Kahn J.S. (2003). Human metapneumovirus infection in the United States: clinical manifestations associated with a newly emerging respiratory infection in children. Pediatrics.

[9] Guerrero-Plata A., Casola A., Garofalo R.P. (2005). Human metapneumovirus induces a profile of lung cytokines distinct from that of respiratory syncytial virus. J. Virol..

[10] Guerrero-Plata A., Casola A., Suarez G., Yu X., Spetch L., Peeples M.E., Garofalo R.P. (2006). Differential response of dendritic cells to human metapneumovirus and respiratory syncytial virus. Am. J. Respir. Cell Mol. Biol..

[11] Bao X., Liu T., Spetch L., Kolli D., Garofalo R.P., Casola A. (2007). Airway epithelial cell response to human metapneumovirus infection. Virology.

[12] Seth R.B., Sun L., Chen Z.J. (2006). Antiviral innate immunity pathways. Cell Res..

[13] Kawai T., Akira S. (2006). TLR signaling. Cell Death. Differ..

[14] Takeda K., Akira S. (2005). Toll-like receptors in innate immunity. Int. Immunol..

[15] Groskreutz D.J., Monick M.M., Powers L.S., Yarovinsky T.O., Look D.C., Hunninghake G.W. (2006). Respiratory syncytial virus induces TLR3 protein and protein kinase R, leading to increased double-stranded RNA responsiveness in airway epithelial cells. J. Immunol..

[16] Liu P., Jamaluddin M., Li K., Garofalo R.P., Casola A., Brasier A.R. (2007). Retinoic Acid-inducible gene I mediates early antiviral response and toll-like receptor 3 expression in respiratory syncytial virus-infected airway epithelial cells. J. Virol..

[17] Liao S., Bao X., Liu T., Lai S., Li K., Garofalo R.P., Casola A. (2008). Role of retinoic acid inducible gene-I in human metapneumovirus-induced cellular signalling. J. Gen. Virol..

[18] Kolli D., Bao X., Liu T., Hong C., Wang T., Garofalo R.P., Casola A. (2011). Human metapneumovirus glycoprotein G inhibits TLR4-dependent signaling in monocyte-derived dendritic cells. J. Immunol..

[19] Beutler B., Hoebe K., Georgel P., Tabeta K., Du X. (2005). Genetic analysis of innate immunity: Identification and function of the TIR adapter proteins. Adv. Exp. Med. Biol..

[20] Haynes L.M., Moore D.D., Kurt-Jones E.A., Finberg R.W., Anderson L.J., Tripp R.A. (2001). Involvement of toll-like receptor 4 in innate immunity to respiratory syncytial virus. J. Virol..

[21] Rassa J.C., Meyers J.L., Zhang Y., Kudaravalli R., Ross S.R. (2002). Murine retroviruses activate B cells via interaction with toll-like receptor 4. Proc. Natl. Acad. Sci. U.S.A..

[22] Okumura A., Pitha P.M., Yoshimura A., Harty R.N. (2010). Interaction between Ebola virus glycoprotein and host toll-like receptor 4 leads to induction of proinflammatory cytokines and SOCS1. J. Virol..

[23] Kurt-Jones E.A., Popova L., Kwinn L., Haynes L.M., Jones L.P., Tripp R.A., Walsh E.E., Freeman M.W., Golenbock D.T., Anderson L.J., Finberg R.W. (2000). Pattern recognition receptors TLR4 and CD14 mediate response to respiratory syncytial virus. Nat. Immunol.

[24] Lizundia R., Sauter K.S., Taylor G., Werling D. (2008). Host species-specific usage of the TLR4-LPS receptor complex. Innate. Immun..

[25] Haeberle H., Takizawa R., Casola A., Brasier A.R., Dieterich H.-J., van Rooijen N., Gatalica Z., Garofalo R.P. (2002). Respiratory syncytial virus-induced activation of NF-kB in the lung involves alveolar macrophages and Toll-like receptor 4-dependent pathways. J. Infect. Dis..

[26] Cyr S.L., Angers I., Guillot L., Stoica-Popescu I., Lussier M., Qureshi S., Burt D.S., Ward B.J. (2009). TLR4 and MyD88 control protection and pulmonary granulocytic recruitment in a murine intranasal RSV immunization and challenge model. Vaccine.

[27] Velayutham T.S., Kolli D., Ivanciuc D., Garofalo R.P., Casola A. (2012). Critical role of Toll-like receptor 4 in human metapneumovirus innate immune responses and disease pathogenesis. J. Infect. Dis..

[28] Heil F., Hemmi H., Hochrein H., Ampenberger F., Kirschning C., Akira S., Lipford G., Wagner H., Bauer S. (2004). Species-specific recognition of single-stranded RNA via toll-like receptor 7 and 8. Science.

[29] Jurk M., Heil F., Vollmer J., Schetter C., Krieg A.M., Wagner H., Lipford G., Bauer S. (2002). Human TLR7 or TLR8 independently confer responsiveness to the antiviral compound R-848. Nat. Immunol..

[30] Krug A., French A.R., Barchet W., Fischer J.A., Dzionek A., Pingel J.T., Orihuela M.M., Akira S., Yokoyama W.M., Colonna M. (2004). TLR9-dependent recognition of MCMV by IPC and DC generates coordinated cytokine responses that activate antiviral NK cell function. Immunity..

[31] Krug A., Luker G.D., Barchet W., Leib D.A., Akira S., Colonna M. (2004). Herpes simplex virus type 1 activates murine natural interferon-producing cells through toll-like receptor 9. Blood.

[32] Goutagny N., Jiang Z., Tian J., Parroche P., Schickli J., Monks B.G., Ulbrandt N., Ji H., Kiener P.A., Coyle A.J., Fitzgerald K.A. (2010). Cell type-specific recognition of human metapneumoviruses (HMPVs) by retinoic acid-inducible gene I (RIG-I) and TLR7 and viral interference of RIG-I ligand recognition by HMPV-B1 phosphoprotein. J. Immunol..

[33] Davidson S., Kaiko G., Loh Z., Lalwani A., Zhang V., Spann K., Foo S.Y., Hansbro N., Uematsu S., Akira S., Matthaei K.I., Rosenberg H.F., Foster P.S., Phipps S. (2011). Plasmacytoid Dendritic Cells Promote Host Defense against Acute Pneumovirus Infection via the TLR7ΓÇôMyD88-Dependent Signaling Pathway. The Journal of Immunology.

[34] Akira S., Uematsu S., Takeuchi O. (2006). Pathogen recognition and innate immunity. Cell.

[35] Yamamoto M., Sato S., Hemmi H., Hoshino K., Kaisho T., Sanjo H., Takeuchi O., Sugiyama M., Okabe M., Takeda K., Akira S. (2003). Role of adaptor TRIF in the MyD88-independent toll-like receptor signaling pathway. Science.

[36] Andrejeva J., Childs K.S., Young D.F., Carlos T.S., Stock N., Goodbourn S., Randall R.E. (2004). The V proteins of paramyxoviruses bind the IFN-inducible RNA helicase, mda-5, and inhibit its activation of the IFN-beta promoter. Proc. Natl. Acad. Sci. U.S.A..

[37] Breiman A., Grandvaux N., Lin R., Ottone C., Akira S., Yoneyama M., Fujita T., Hiscott J., Meurs E.F. (2005). Inhibition of RIG-I-dependent signaling to the interferon pathway during hepatitis C virus expression and restoration of signaling by IKKepsilon. J. Virol..

[38] tenOever B.R., Sharma S., Zou W., Sun Q., Grandvaux N., Julkunen I., Hemmi H., Yamamoto M., Akira S., Yeh W.C., Lin R., Hiscott J. (2004). Activation of TBK1 and IKKvarepsilon kinases by vesicular stomatitis virus infection and the role of viral ribonucleoprotein in the development of interferon antiviral immunity. J. Virol..

[39] Siren J., Imaizumi T., Sarkar D., Pietila T., Noah D.L., Lin R., Hiscott J., Krug R.M., Fisher P.B., Julkunen I., Matikainen S. (2006). Retinoic acid inducible gene-I and mda-5 are involved in influenza A virus-induced expression of antiviral cytokines. Microbes. Infect..

[40] Pichlmair A., Schulz O., Tan C.P., Naslund T.I., Liljestrom P., Weber F., Reis e S. (2006). RIG-I-mediated antiviral responses to single-stranded RNA bearing 5'-phosphates. Science.

[41] Hiscott J., Lin R., Nakhaei P., Paz S. (2006). MasterCARD: a priceless link to innate immunity. Trends Mol. Med..

[42] Johnson C.L., Gale M. (2006). CARD games between virus and host get a new player. Trends Immunol..

[43] Kawai T., Takahashi K., Sato S., Coban C., Kumar H., Kato H., Ishii K.J., Takeuchi O., Akira S. (2005). IPS-1, an adaptor triggering RIG-I- and Mda5-mediated type I interferon induction. Nat. Immunol..

[44] Meylan E., Tschopp J. (2006). Toll-like receptors and RNA helicases: two parallel ways to trigger antiviral responses. Mol. Cell.

[45] Liu Y.J., Kanzler H., Soumelis V., Gilliet M. (2001). Dendritic cell lineage, plasticity and cross-regulation. Nat. Immunol..

[46] Rinaldo C.R., Piazza P. (2004). Virus infection of dendritic cells: portal for host invasion and host defense. Trends Microbiol..

[47] Stumbles P.A., Upham J.W., Holt P.G. (2003). Airway dendritic cells: co-ordinators of immunological homeostasis and immunity in the respiratory tract. APMIS.

[48] Rescigno M., Borrow P. (2001). The host-pathogen interaction: new themes from dendritic cell biology. Cell.

[49] Tan M.C., Battini L., Tuyama A.C., Macip S., Melendi G.A., Horga M.A., Gusella G.L. (2007). Characterization of human metapneumovirus infection of myeloid dendritic cells. Virology.

[50] Le N.C., Munir S., Losq S., Winter C.C., McCarty T., Stephany D.A., Holmes K.L., Bukreyev A., Rabin R.L., Collins P.L., Buchholz U.J. (2009). Infection and maturation of monocyte-derived human dendritic cells by human respiratory syncytial virus, human metapneumovirus, and human parainfluenza virus type 3. Virology.

[51] Guerrero-Plata A., Baron S., Poast J.S., Adegboyega P.A., Casola A., Garofalo R.P. (2005). Activity and regulation of alpha interferon in respiratory syncytial virus and human metapneumovirus experimental infections. J. Virol..

[52] Horvath C.M. (2004). Silencing STATs: lessons from paramyxovirus interferon evasion. Cytokine Growth Factor Rev..

[53] Horvath C.M. (2004). Weapons of STAT destruction. Interferon evasion by paramyxovirus V protein. Eur. J. Biochem..

[54] Bao X., Liu T., Spetch L., Kolli D., Garofalo R.P., Casola A. (2007). Airway epithelial cell response to human metapneumovirus infection. Virology.

[55] Colamonici O.R., Uyttendaele H., Domanski P., Yan H., Krolewski J.J. (1994). p135tyk2, an interferon-alpha-activated tyrosine kinase, is physically associated with an interferon-alpha receptor. J. Biol. Chem..

[56] Colamonici O., Yan H., Domanski P., Handa R., Smalley D., Mullersman J., Witte M., Krishnan K., Krolewski J. (1994). Direct binding to and tyrosine phosphorylation of the alpha subunit of the type I interferon receptor by p135tyk2 tyrosine kinase. Mol. Cell Biol..

[57] Leung S., Qureshi S.A., Kerr I.M., Darnell J.E., Stark G.R. (1995). Role of STAT2 in the alpha interferon signaling pathway. Mol. Cell Biol..

[58] Qureshi S.A., Salditt-Georgieff M., Darnell J.E. (1995). Tyrosine-phosphorylated Stat1 and Stat2 plus a 48-kDa protein all contact DNA in forming interferon-stimulated-gene factor 3. Proc. Natl. Acad. Sci. U.S.A..

[59] Uze G., Schreiber G., Piehler J., Pellegrini S. (2007). The receptor of the type I interferon family. Curr. Top. Microbiol. Immunol..

[60] Li X., Leung S., Burns C., Stark G.R. (1998). Cooperative binding of Stat1-2 heterodimers and ISGF3 to tandem DNA elements. Biochimie.

[61] Hata N., Sato M., Takaoka A., Asagiri M., Tanaka N., Taniguchi T. (2001). Constitutive IFN-alpha/beta signal for efficient IFN-alpha/beta gene induction by virus. Biochem. Biophys. Res. Commun..

[62] Trinchieri G. (2010). Type I interferon: friend or foe?. J. Exp. Med..

[63] Hervas-Stubbs S., Perez-Gracia J.L., Rouzaut A., Sanmamed M.F., Le B.A., Melero I. (2011). Direct effects of type I interferons on cells of the immune system. Clin. Cancer Res..

[64] Christophi G.P., Hudson C.A., Panos M., Gruber R.C., Massa P.T. (2009). Modulation of macrophage infiltration and inflammatory activity by the phosphatase SHP-1 in virus-induced demyelinating disease. J. Virol..

[65] Yasukawa H., Misawa H., Sakamoto H., Masuhara M., Sasaki A., Wakioka T., Ohtsuka S., Imaizumi T., Matsuda T., Ihle J.N., Yoshimura A. (1999). The JAK-binding protein JAB inhibits Janus tyrosine kinase activity through binding in the activation loop. EMBO J..

[66] Ramaswamy M., Shi L., Monick M.M., Hunninghake G.W., Look D.C. (2004). Specific inhibition of type I interferon signal transduction by respiratory syncytial virus. Am. J. Respir. Cell Mol. Biol..

[67] Didcock L., Young D.F., Goodbourn S., Randall R.E. (1999). The V protein of simian virus 5 inhibits interferon signalling by targeting STAT1 for proteasome-mediated degradation. J. Virol..

[68] Kubota T., Yokosawa N., Yokota S., Fujii N. (2001). C terminal CYS-RICH region of mumps virus structural V protein correlates with block of interferon alpha and gamma signal transduction pathway through decrease of STAT 1-alpha. Biochem. Biophys. Res. Commun..

[69] Rodriguez J.J., Cruz C.D., Horvath C.M. (2004). Identification of the nuclear export signal and STAT-binding domains of the Nipah virus V protein reveals mechanisms underlying interferon evasion. J. Virol..

[70] Rodriguez J.J., Parisien J.P., Horvath C.M. (2002). Nipah virus V protein evades alpha and gamma interferons by preventing STAT1 and STAT2 activation and nuclear accumulation. J. Virol..

[71] Takeuchi K., Kadota S.I., Takeda M., Miyajima N., Nagata K. (2003). Measles virus V protein blocks interferon (IFN)-alpha/beta but not IFN-gamma signaling by inhibiting STAT1 and STAT2 phosphorylation. FEBS Lett..

[72] Dinwiddie D.L., Harrod K.S. (2008). Human Metapneumovirus Inhibits IFN-{alpha} Signaling Through Inhibition of STAT1 Phosphorylation. Am. J. Respir. Cell Mol. Biol..

[73] Ren J., Kolli D., Liu T., Xu R., Garofalo R.P., Casola A., Bao X. (2011). Human metapneumovirus inhibits IFN-beta signaling by downregulating Jak1 and Tyk2 cellular levels. PLoS. ONE..

[74] Bao X., Kolli D., Liu T., Shan Y., Garofalo R.P., Casola A. (2008). Human metapneumovirus small hydrophobic protein inhibits NF-kappaB transcriptional activity. J. Virol..

[75] Bao X., Liu T., Shan Y., Li K., Garofalo R.P., Casola A. (2008). Human metapneumovirus glycoprotein G inhibits innate immune responses. PLoS. Pathog..

[76] Ren J., Wang Q., Kolli D., Prusak D.J., Tseng C.T., Chen Z.J., Li K., Wood T.G., Bao X. (2012). Human metapneumovirus M2-2 protein inhibits innate cellular signaling by targeting MAVS. J. Virol..

[77] van den Hoogen B.G., Bestebroer T.M., Osterhaus A.D., Fouchier R.A. (2002). Analysis of the genomic sequence of a human metapneumovirus. Virology.

[78] Biacchesi S., Skiadopoulos M.H., Boivin G., Hanson C.T., Murphy B.R., Collins P.L., Buchholz U.J. (2003). Genetic diversity between human metapneumovirus subgroups. Virology.

[79] Biacchesi S., Murphy B.R., Collins P.L., Buchholz U.J. (2007). Frequent frameshift and point mutations in the SH gene of human metapneumovirus passaged in vitro. J. Virol..

[80] Biacchesi S., Skiadopoulos M.H., Yang L., Lamirande E.W., Tran K.C., Murphy B.R., Collins P.L., Buchholz U.J. (2004). Recombinant human Metapneumovirus lacking the small hydrophobic SH and/or attachment G glycoprotein: deletion of G yields a promising vaccine candidate. J. Virol..

[81] Biacchesi S., Pham Q.N., Skiadopoulos M.H., Murphy B.R., Collins P.L., Buchholz U.J. (2005). Infection of nonhuman primates with recombinant human metapneumovirus lacking the SH, G, or M2-2 protein categorizes each as a nonessential accessory protein and identifies vaccine candidates. J. Virol..

[82] Wilson R.L., Fuentes S.M., Wang P., Taddeo E.C., Klatt A., Henderson A.J., He B. (2006). Function of small hydrophobic proteins of paramyxovirus. J. Virol..

[83] Fuentes S., Tran K.C., Luthra P., Teng M.N., He B. (2007). Function of the Respiratory Syncytial Virus small hydrophobic protein. J. Virol..

[84] Li Z., Xu J., Patel J., Fuentes S., Lin Y., Anderson D., Sakamoto K., Wang L.F., He B. (2011). Function of the small hydrophobic protein of J paramyxovirus. J. Virol..

[85] Xu P., Li Z., Sun D., Lin Y., Wu J., Rota P.A., He B. (2011). Rescue of wild-type mumps virus from a strain associated with recent outbreaks helps to define the role of the SH ORF in the pathogenesis of mumps virus. Virology.

[86] Karin M., Delhase M. (2000). The I kappa B kinase (IKK) and NF-kappa B: key elements of proinflammatory signalling. Semin. Immunol.

[87] Schmidt M., Chiorini J.A., Afione S., Kotin R. (2002). Adeno-associated virus type 2 Rep78 inhibition of PKA and PRKX: fine mapping and analysis of mechanism. J. Virol..

[88] Schowalter R.M., Smith S.E., Dutch R.E. (2006). Characterization of human metapneumovirus F protein-promoted membrane fusion: critical roles for proteolytic processing and low pH. J. Virol..

[89] Cox R.G., Livesay S.B., Johnson M., Ohi M.D., Williams J.V. (2012). The Human Metapneumovirus Fusion Protein Mediates Entry Via an Interaction with RGD-binding Integrins. J. Virol..

[90] Kolli D., Bao X., Liu T., Hong C., Wang T., Garofalo R.P., Casola A. (2011). Human metapneumovirus glycoprotein G inhibits TLR4-dependent signaling in monocyte-derived dendritic cells. J. Immunol..

[91] Bao X., Kolli D., Ren J., Liu T., Garofalo R.P., Casola A. (2012). Human Metapneumovirus Glycoprotein G Targets RIG-I to Inhibit Airway Epithelial Cell Responses. J. Virol..

[92] Pulendran B., Palucka K., Banchereau J. (2001). Sensing pathogens and tuning immune responses. Science.

[93] Mellman I., Steinman R.M. (2001). Dendritic cells: Specialized and regulated antigen processing machines. Cell.

[94] Delgado M.F., Coviello S., Monsalvo A.C., Melendi G.A., Hernandez J.Z., Batalle J.P., Diaz L., Trento A., Chang H.Y., Mitzner W., Ravetch J., Melero J.A., Irusta P.M., Polack F.P. (2009). Lack of antibody affinity maturation due to poor Toll-like receptor stimulation leads to enhanced respiratory syncytial virus disease. Nat. Med..

[95] Polack F.P., Irusta P.M., Hoffman S.J., Schiatti M.P., Melendi G.A., Delgado M.F., Laham F.R., Thumar B., Hendry R.M., Melero J.A., Karron R.A., Collins P.L., Kleeberger S.R. (2005). The cysteine-rich region of respiratory syncytial virus attachment protein inhibits innate immunity elicited by the virus and endotoxin. Proc. Natl. Acad. Sci. U.S.A..

[96] Arnold R., Konig B., Werchau H., Konig W. (2004). Respiratory syncytial virus deficient in soluble G protein induced an increased proinflammatory response in human lung epithelial cells. Virology.

[97] Alff P.J., Gavrilovskaya I.N., Gorbunova E., Endriss K., Chong Y., Geimonen E., Sen N., Reich N.C., Mackow E.R. (2006). The pathogenic NY-1 hantavirus G1 cytoplasmic tail inhibits RIG-I- and TBK-1-directed interferon responses. J. Virol..

[98] Geimonen E., LaMonica R., Springer K., Farooqui Y., Gavrilovskaya I.N., Mackow E.R. (2003). Hantavirus pulmonary syndrome-associated hantaviruses contain conserved and functional ITAM signaling elements. J. Virol..

[99] Buchholz U.J., Biacchesi S., Pham Q.N., Tran K.C., Yang L., Luongo C.L., Skiadopoulos M.H., Murphy B.R., Collins P.L. (2005). Deletion of M2 gene open reading frames 1 and 2 of human metapneumovirus: Effects on RNA synthesis, attenuation, and immunogenicity. J. Virol..

[100] Herfst S., de Graaf M., Schickli J.H., Tang R.S., Kaur J., Yang C.F., Spaete R.R., Haller A.A., van den Hoogen B.G., Osterhaus A.D., Fouchier R.A. (2004). Recovery of human metapneumovirus genetic lineages a and B from cloned cDNA. J. Virol..

[101] Sutherland K.A., Collins P.L., Peeples M.E. (2001). Synergistic effects of gene-end signal mutations and the M2-1 protein on transcription termination by respiratory syncytial virus. Virology.

[102] Schickli J.H., Kaur J., MacPhail M., Guzzetta J.M., Spaete R.R., Tang R.S. (2008). Deletion of human metapneumovirus M2-2 increases mutation frequency and attenuates growth in hamsters. Virol. J..

[103] Ren J., Wang Q., Kolli D., Prusak D.J., Tseng C.T., Li K., Wood T.G., Bao X. (2012). Human metapneumovirus M2-2 protein inhibits the innate cellular signaling by targeting MAVS. J. Virol..

[104] Guo Z., Chen L.M., Zeng H., Gomez J.A., Plowden J., Fujita T., Katz J.M., Donis R.O., Sambhara S. (2007). NS1 protein of influenza A virus inhibits the function of intracytoplasmic pathogen sensor, RIG-I. Am. J. Respir. Cell Mol. Biol..

[105] Opitz B., Rejaibi A., Dauber B., Eckhard J., Vinzing M., Schmeck B., Hippenstiel S., Suttorp N., Wolff T. (2007). IFNbeta induction by influenza A virus is mediated by RIG-I which is regulated by the viral NS1 protein. Cell Microbiol..

[106] Varga Z.T., Ramos I., Hai R., Schmolke M., Garcia-Sastre A., Fernandez-Sesma A., Palese P. (2011). The Influenza Virus Protein PB1-F2 Inhibits the Induction of Type I Interferon at the Level of the MAVS Adaptor Protein. PLoS. Pathog..

[107] Graef K.M., Vreede F.T., Lau Y.F., McCall A.W., Carr S.M., Subbarao K., Fodor E. (2010). The PB2 subunit of the influenza virus RNA polymerase affects virulence by interacting with the mitochondrial antiviral signaling protein and inhibiting expression of beta interferon. J. Virol..

[108] Ling Z., Tran K.C., Teng M.N. (2009). Human respiratory syncytial virus nonstructural protein NS2 antagonizes the activation of beta interferon transcription by interacting with RIG-I. J. Virol..

[109] Ren J., Liu T., Pang L., Li K., Garofalo R.G., Casola A., Bao X. (2011). A Novel Mechanism for Inhibition of IRF-3-Dependent Gene Expression by Human Respiratory Syncytial Virus NS1 Protein. J. Gen. Virol..

[110] Bastien N., Normand S., Taylor T., Ward D., Peret T.C., Boivin G., Anderson L.J., Li Y. (2003). Sequence analysis of the N, P, M and F genes of Canadian human metapneumovirus strains. Virus Res..

[111] Tedcastle A.B., Fenwick F., Ingram R.E., King B.J., Robinson M.J., Toms G.L. (2012). The characterization of monoclonal antibodies to human metapneumovirus and the detection of multiple forms of the virus nucleoprotein and phosphoprotein. J. Med. Virolog..

[112] Derdowski A., Peters T.R., Glover N., Qian R., Utley T.J., Burnett A., Williams J.V., Spearman P., Crowe J.E. (2008). Human metapneumovirus nucleoprotein and phosphoprotein interact and provide the minimal requirements for inclusion body formation. J. Gen. Virol..

